# GDockScore: a graph-based protein–protein docking scoring function

**DOI:** 10.1093/bioadv/vbad072

**Published:** 2023-06-12

**Authors:** Matthew McFee, Philip M Kim

**Affiliations:** Department of Molecular Genetics, The University of Toronto, Toronto, ON M5S 1A8, Canada; Donnelly Centre for Cellular and Biomolecular Research, The University of Toronto, Toronto, ON M5S 3E1, Canada; Department of Molecular Genetics, The University of Toronto, Toronto, ON M5S 1A8, Canada; Donnelly Centre for Cellular and Biomolecular Research, The University of Toronto, Toronto, ON M5S 3E1, Canada; Department of Computer Science, The University of Toronto, Toronto, ON M5S 2E4, Canada

## Abstract

**Summary:**

Protein complexes play vital roles in a variety of biological processes, such as mediating biochemical reactions, the immune response and cell signalling, with 3D structure specifying function. Computational docking methods provide a means to determine the interface between two complexed polypeptide chains without using time-consuming experimental techniques. The docking process requires the optimal solution to be selected with a scoring function. Here, we propose a novel graph-based deep learning model that utilizes mathematical graph representations of proteins to learn a scoring function (GDockScore). GDockScore was pre-trained on docking outputs generated with the Protein Data Bank biounits and the RosettaDock protocol, and then fine-tuned on HADDOCK decoys generated on the ZDOCK Protein Docking Benchmark. GDockScore performs similarly to the Rosetta scoring function on docking decoys generated using the RosettaDock protocol. Furthermore, state-of-the-art is achieved on the CAPRI score set, a challenging dataset for developing docking scoring functions.

**Availability and implementation:**

The model implementation is available at https://gitlab.com/mcfeemat/gdockscore.

**Supplementary information:**

Supplementary data are available at *Bioinformatics Advances* online.

## 1 Introduction

Protein–protein complexes play a vital role in biological processes, such as mediating biochemical reactions, signal transduction and the immune response (Marsh and Teichmann, 2015; Rebsamen *et al.*, 2013). The structures of these complexes are key to their biochemical function (Sowmya *et al.*, 2015) and structural biologists have been using techniques, such as X-ray crystallography, nuclear magnetic resonance spectroscopy and cryogenic electron microscopy (Curry, 2015) to uncover them. However, these techniques are often difficult and time consuming (Nanev, 2020), spurring the development of *in silico* methods to accomplish a variety of tasks involving protein structure (Chevalier *et al.*, 2017; Maia *et al.*, 2020; Sesterhenn *et al.*, 2020; Vakser, 2014). Protein–protein docking is a two staged process where the initial stage involves sampling potential interfaces from the vast complex conformational space, and the second stage involves scoring of potential conformations (decoys) such that near-native interfaces are assigned favourable scores. This stage of the docking process is dependent on scoring functions, which assess the energetic favourability of the proposed decoys (Kastritis and Bonvin, 2011; Moal *et al.*, 2013).

Protein–protein docking involves sampling the immense complex conformational space with the goal of determining the native interface between two polypeptide chains of interest. The conformational sampling process can be divided into three distinct search strategies: exhaustive global searching, local shape feature matching and randomized searching (Huang, 2014). In exhaustive global searching, one protein is held fixed while the rotational and translational degrees of freedom are explored by the other protein, this computationally intensive process can be expedited by applying Fast Fourier Transform techniques (Katchalski-Katzir *et al.*, 1992). In local feature mapping techniques, the structures of the proteins can be represented as a Connolly surface and local complementarity of shapes is found between the two surfaces (Kuntz *et al.*, 1982) or through geometric hashing where local matches in shape predictors are used to determine complementarity (Schneidman-Duhovny *et al.*, 2005). Finally, in randomized searching one protein is randomly placed around the other (often with some constraint for the placements). The placement can then be refined using stochastic processes, such as Markov Monte Carlo Chains. After creating a set of candidate docking decoys, the second stage of the docking process begins. In this process, decoys are scored based on their biophysical favourability using a scoring function.

Scoring functions for protein–protein docking can be placed into four distinct categories including force fields, empirical functions, knowledge-based functions and machine learning-based functions (Huang *et al.*, 2010). Force fields involve calculations of physical atomic interactions, such as van der Waals interactions and electrostatics (Harrison *et al.*, 2018). In empirical scoring functions, energy terms, such as van der Waals and hydrogen bonds, are summed using learned weighting parameters (Pason and Sotriffer, 2016). Knowledge-based scoring functions employ statistical potentials to score interactions (Gohlke *et al.*, 2000). Finally, machine learning-based scoring functions leverage the increasing availability of protein complex structural data to learn scoring functions that can potentially have millions of parameters without the feature engineering involved in many traditional development techniques.

Previously, classical machine-learning techniques, such as support vector machines (Basu and Wallner, 2016; Kinnings *et al.*, 2011) and random forests (Li *et al.*, 2015; Zilian and Sotriffer, 2013), were used to develop scoring functions. However, these techniques still require feature engineering and are less performant than deep learning models. In deep learning, two types of network architectures have been used prominently to develop scoring functions. Convolutional neural networks have been used by discretizing the protein structure and generating 3D feature grids that are then passed through 3D convolutional layers (McNutt *et al.*, 2021; Renaud *et al.*, 2021; Schneider *et al.*, 2022; Wang *et al.*, 2019). Secondly, graph convolutional networks have been used where proteins are represented as mathematical graphs and node information is shared through message passing to learn a meaningful graph embedding (Cao and Shen, 2020; Geng *et al.*, 2020; Strokach *et al.*, 2020; Wang *et al.*, 2021). The use of convolutional layers is problematic in that they typically learn local information across the protein representation and cannot handle long distance relationships. Although there has been success in the use of dilated convolutional layers to learn relationships across greater distances in sequence data (Fudenberg *et al.*, 2020; Yu and Koltun, 2016). Furthermore, while translationally invariant, convolutional networks are not invariant to rotations of input data. Finally, these networks require voxelization of the interface of interest (discretization of the interface into a 3D, image-like grid), which may result in loss of important information (Wang *et al.*, 2021).

Our proposed scoring function utilizes a protein graph representation that has previously been very effective in learning embeddings for protein design and peptide binding prediction (Abdin *et al.*, 2022; Ingraham *et al.*, 2019). This type of representation resolves the issues in standard convolutional networks by avoiding discretization, and being rotationally and translationally invariant. The protein graph embedding technique was further extended to learn graph embeddings of multimeric proteins, which was done by developing a method to exchange interface information bi-directionally between protein graphs during the learning process. We show the effectiveness of our model architecture by learning a model pre-trained on RosettaDock (Roy Burman *et al.*, 2018) generated on the Protein Data Bank (PDB) biounits, and fine-tuned on HADDOCK decoys (Dominguez *et al.*, 2003; Renaud *et al.*, 2021) using the ZDOCK Protein Benchmark dataset (Vreven *et al.*, 2015). In this way, we allowed the model to train on a more diverse set of interfaces, the PDB RosettaDock decoys, as well as highly refined HADDOCK decoys, which provide information about backbone flexibility. Our model performs well on RosettaDock decoys and on a gold-standard protein–protein docking scoring function development set known as the CAPRI score set (Lensink and Wodak, 2014).

## 2 Results

### 2.1 GDockScore is a novel architecture utilizing protein graph attention and bi-directional interface information exchange

The model architecture utilized in GDockScore builds upon the parallel embedding architecture utilized by Abdin *et al.* (2022) and protein graph attention developed by Ingraham *et al.* (2019). Details of the model input representation are available in Section 4. The model architecture is as follows: after an initial embedding of input node and edge matrices with linear layers, the basic building block of the architecture includes a protein graph attention block (Ingraham *et al.*, 2019) that updates the node embedding for each protein residue. In these blocks, the edge matrices pass through linear layers to generate key and value pairs, and the node features are used to generate query vectors. The attention operation is then performed as seen in the original presentation of Transformers (Vaswani *et al.*, 2017) with the modification that only the 30 nearest neighbours to each node in the graph are used in the update to improve computational efficiency. As the Ingraham *et al.* protein graph attention is designed to update a protein graph embedding in isolation, we further developed a means of exchanging interfacial information between the two protein graph embeddings known as the bi-directional exchange layer.

**Figure 1.**
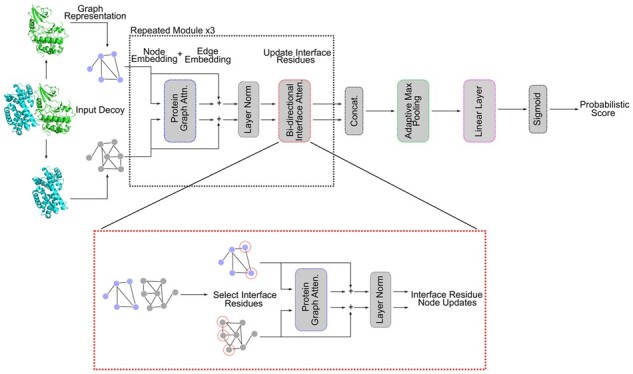
The model architecture described in this article. The inlaid image (dotted red line) illustrates the bi-directional exchange of information between protein graph embeddings. The protein depicted is the AB interface from PDB code 1f5q’s biological assembly 1. Module border colours indicate module type

In the bi-directional exchange layer, the rows in the node embedding of the protein graph corresponding to interfacial residues are extracted into a separate matrix, hereby referred to as the interface embedding matrix. This matrix is used to generate a query matrix, while key and value pairs are generated from a matrix consisting of edge vectors from the interfacial residues of the current graph embedding to the 10 nearest neighbours on the other protein chain in the complex. As in previous models (Abdin *et al.*, 2022; Ingraham *et al.*, 2019), the corresponding node vectors on the adjacent proteins are concatenated to the appropriate edge vectors. This concatenation allows each protein embedding to directly ‘see’ the residues of the other protein via node features. The neighbour-based attention mechanism previously discussed is then performed to generate an update to each of the interface residue embeddings. The interface residue embeddings in the node matrix for the entire protein are then replaced with these updated interfacial residue node embeddings. This process is then repeated in the other direction to update the other protein chain in the complex. This basic module is stacked multiple times to generate final protein embeddings for each protein which are then concatenated and pooled down to produce a final output, which passes through the sigmoid function to generate a probability of being a near-native decoy. Mathematical details of this layer are provided in Section 4 and the overall model architecture is illustrated in Figure 1.

### 2.2 Generation of a novel protein–protein docking pre-training dataset leveraging the PDB biounit files for model pre-training

Previously, protein–protein docking deep learning models were trained on gold-standard datasets, such as the ZDOCK protein–protein docking benchmark (Hwang *et al.*, 2010), and Dockground (Kundrotas *et al.*, 2018). These datasets rely on decoys generated for a small number of targets and thus do not leverage the full complexity of structures and interfaces exhibited in the PDB. We generated a significantly more varied dataset for model pre-training by scanning all PDB biounit files for interacting chain pairs (snapshot March 2021) to re-dock to form interfaces of varying quality. The PDB biounits are the proposed functional forms of the relevant models and may consist of one or more X-ray crystallography asymmetric units. Thus, the structures being used are in the bound state.

**Figure 2.**
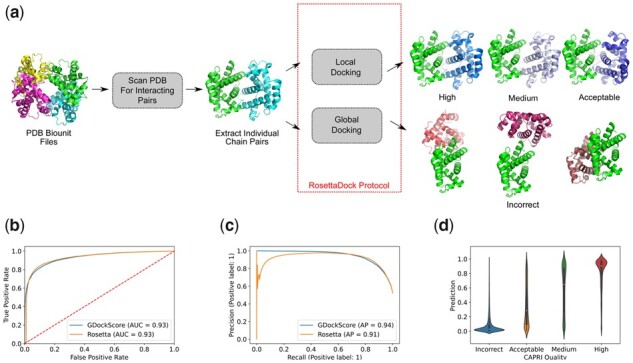
(**a**) Schematic illustration of the data generation process using the PDB biounit files used to create the model pre-training set. The biological assembly 1 for PDB code 1bab is illustrated. (**b**) The ROC (with AUC) for our model in comparison to Rosetta’s interface score (I_sc). *P*-value 0.3068. (**c**) The precision–recall curves for our model versus I_sc with AP indicated. The one sample *t*-test *P*-value for re-sampled differences in AP is 0.0 according to the SciPy Welch’s *t*-test. (**d**) Violin plot showing the distribution of predictions for each CAPRI quality using our models output

To generate this pre-training set, we searched for biounit complexes that had at least three pairs of amino acids with an *α*-carbon to *α*-carbon distance of 6 Å or less and then performed local and global dockings with RosettaDock, which produced interfaces of varying quality. The CAPRI standards were used to indicate the quality of the decoys. These standards classify decoys into qualities including incorrect, acceptable, medium and high, in order of increasing near-nativeness (Méndez *et al.*, 2003). The CAPRI quality metric uses measurable quantities, such as interface root-mean-square deviation (iRMSD), and the fraction of contacts in the true interface recapitulated in the proposed decoy to assign the quality of the model. The decoys used to train our model were generated by using the RosettaDock protocol (Roy Burman *et al.*, 2018). For positive examples, the ligand protein was initialized to a position near the true interface with a small perturbation allowing for the ligand to re-dock to the receptor protein in a near-native configuration. This methodology typically generated interfaces of acceptable or better quality. To generate the negative dataset, the ligand protein initial position was completely randomized around the receptor protein and the receptor was also rotated randomly. The RosettaDock protocol was then carried out, generating completely new interfaces with CAPRI quality incorrect, indicating a significant difference from the native interface. This data generation process is depicted in Figure 2a.

The above data generation protocol resulted in significantly more negative docks being generated in comparison to CAPRI quality acceptable or better examples. This is due to many chain pairs failing to generate CAPRI acceptable decoys even when initialized very close to the true interface and docked with RosettaDock as well as the algorithm generally being able to recover a solution very close to the true interface in the local docking protocol. To balance the data, a subset of CAPRI incorrect, and acceptable or better decoys were sampled such that the total number of positive examples (acceptable, medium and high) equalled the negative examples. Due to the generation process, there were roughly an equal number of high quality decoys to the total number of acceptable and medium. We kept this imbalance as we want to favour protein interfaces that are as close to the optimal interface as possible. To ensure no significant similarity between the training and validation sets, both sequence and structural analysis were performed, for details consult Section 4.

The standard RosettaDock protocol only adjusts side-chain conformations at the interface and does not predict backbone conformational changes. Based on initial model analysis, we hypothesized the model could be supplemented with additional data. The DeepRank (Renaud *et al.*, 2021) dataset includes HADDOCK (Dominguez *et al.*, 2003) docking decoys generated using the ZDOCK Protein Docking Benchmark (BM5.5) (Vreven *et al.*, 2015) dataset. We exclusively selected the decoys that had backbone refinement performed at the interface (‘it1’ decoys) and those further refined with a short molecular dynamics simulation in water (‘itw’ decoys). In this set all decoys with an iRMSD ≤4 were considered positive examples. The pre-trained model parameters were then fine-tuned with this dataset.

### 2.3 Generation of a novel protein–protein docking pre-training dataset leveraging the PDB biounit files for model pre-training

First, we sought to assess the model’s performance on a test set consisting of decoys generated with our pre-training dataset generation scheme. For each decoy, the Rosetta interface score (I_sc) was also computed, with the I_sc being the difference in Rosetta arbitrary energy units of the separated chains, and the bound chains. When docking proteins with RosettaDock, this interface energy is used to rank the decoys. The final area under the curve (AUC) achieved by the trained model on the test set was 0.93 indicating that the model effectively learned to classify unseen protein-protein docking decoys with structures and sequences differing from those seen in the training set (Fig. 2b). The fine-tuning on the HADDOCK decoys does not appear to significantly impact GDockScore’s ability to classify RosettaDock outputs. Furthermore, the average precision (AP) as determined by the sklearn package (Pedregosa *et al.*, 2011) for the model was 0.94 (Fig. 2c). We sought to directly compare the performance of the learned model to the classical Rosetta scoring function (Alford *et al.*, 2017). In comparison to our learned model, Rosetta achieved 0.93 AUC on the test set (Fig. 2b). However, according to the DeLong statistical test (DeLong *et al.*, 1988), which compares if AUC of the ROC (AUCROC) values are statistically different, the *P*-value of this difference is only 0.3068, greater than our significance cut-off α=0.05. Thus, it appears that our model at least learns to recapitulate the Rosetta scoring function. This suggests that fine-tuning on the HADDOCK decoys does not significantly reduce the models ability to score RosettaDock decoys. More interestingly, when comparing the AP, see Equation (3) in Section 4 for details, our model outperforms Rosetta. Rosetta achieves an AP of 0.91 compared to our AP of 0.94. Using re-sampling to determine the difference in AP for the two models indicates a statistically significant difference in AP values with a *P*-value of 0.0 as reported by SciPy. This indicates that our model typically achieves a greater precision in comparison to the standard Rosetta scoring function.

To better understand how the model was performing on individual CAPRI qualities, the model predictions for each quality were plotted as a violin plot (Fig. 2d). The model appears to confidently predict high quality and incorrect models as indicated by the majority of the violin plot’s density being focussed at very high and very low scores. The high quality models are very close to the native interface with iRMSD values ≤1 and the decoys, which are incorrect according to CAPRI standards will have very large iRMSD values >4. Intuitively, the model appears to struggle more on CAPRI acceptable and medium. Therefore, it can be concluded that our model can confidently determine an almost exact interface from a completely incorrect one but it does struggle with lower quality models that are still somewhat near the true interface i.e. models categorized as acceptable. This is expected, as the acceptable models can have iRMSDs as high as four, which indicates a substantial deviation from the true interface and even small rotations and translations may result in unfavourable amino acid contacts being made at the decoy interface.

As a final check of the model in predicting high quality interfaces, we applied our model to the new, but related task of crystal and biological interface detection. Crystal interfaces are protein–protein interfaces that appear during the generation of protein crystals in X-ray crystallography experiments, which are not biologically relevant. The MANY (Baskaran *et al.*, 2014) and DC (Duarte *et al.*, 2012) datasets of real biological and crystal interfaces were combined and scored by the model. The model achieved an AUCROC of 0.93 and AP 0.92 on the precision–recall curve, indicating strong performance on differentiating crystal interfaces from biological interfaces even though the model was trained on protein–protein docking decoys (Supplementary Fig. S1a and b). The model is more confident at classifying the biological interfaces than the crystal (Supplementary Fig. S1c). These performance metrics are likely biased as there is sequence redundancy in the training set. However, this result is still promising in showing that the model architecture can easily be extended to similar tasks to protein–protein docking.

### 2.4 GDockScore outperforms state-of-the-art models on the challenging CAPRI score set

We sought to test our model on an externally generated gold-standard test set to verify its performance. We used the CAPRI score set (Lensink and Wodak, 2014), a dataset for developing protein–protein docking scoring functions. This dataset consists of decoys generated for 13 unique chain pairs (predominantly the unbound structures) generated by 47 unique docking methods and is noted for being challenging. The version of the CAPRI score set used in this article was the copy with improved PDB formatting, and short HADDOCK energy function refinements, used in the development of iScore and DeepRank (Geng *et al.*, 2020; Renaud *et al.*, 2021). We benchmarked our model against DeepRank (Renaud *et al.*, 2021) and GNN-DOVE (Wang *et al.*, 2021), two recently developed deep learning models for interface scoring.

**Figure 3.**
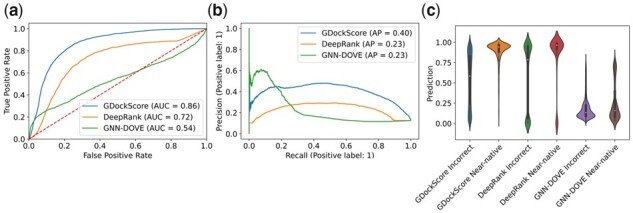
(**a**) The ROC for GDockScore, DeepRank and GNN-DOVE where AUCs are indicated in the inlaid figure legend and the red dotted line indicates luck. All pair-wise comparisons have *P*-value <2.2e−16. (**b**) The precision–recall curves of our model, DeepRank, and GNN-DOVE with AP indicated in the inlayed legend. The pair-wise Welch’s *t*-test values obtained via re-sampling are 0.0 for GDockScore and GNN-DOVE, 0.0 for GDockScore and DeepRank and 0.2106 for DeepRank and GNN-DOVE. (**c**) Violin plot of each model’s predictions for near-native versus incorrect models. Note: DeepRank predicts the probability of the model being incorrect so we have taken one minus this probability so that each model output is in agreement

We sought to compare our model against GNN-DOVE and DeepRank using the benchmark of AUC of the ROC, AP of the precision–recall curve, as well as comparing violin plots of score distributions for near-native versus incorrect decoys. In terms of the AUCROC, our model performs better than both DeepRank and GNN-DOVE with an AUCROC of 0.86 compared to 0.72 and 0.54, respectively (Fig. 3a). Again, using the DeLong test (DeLong *et al.*, 1988), all pair-wise differences have a *P*-value of <2e-16, indicating statistically different AUCROC values even after Bonferonni correction. It is important to note that the initial model without any fine-tuning on the DeepRank dataset only achieves an AUC of 0.72, indicating that our fine-tuning set is providing significant useful information to GDockScore. Our model also achieves an improved AP in comparison to these two models, achieving 0.40 in comparison to 0.23 for DeepRank, and 0.23 for GNN-DOVE (Fig. 3b). Comparing differences in AP for the PR curves for each model using re-sampling revealed statistically significant differences with *P*-values of 0.0 when comparing GDockScore to GNN-DOVE and DeepRank, and a *P*-value of 0.2106 when comparing GNN-DOVE to DeepRank. The model with no fine-tuning achieves an AP of 0.19. To determine how well the models were differentiating the two classes of near-native and incorrect interfaces, a violin plot was generated (Fig. 3c). GDockScore seems to do a slightly better job at applying lower scores to the incorrect decoys, whereas DeepRank likes to score most decoys in the CAPRI score set highly. Our model also seems to provide higher scores for the native class, as indicated by increasing density at higher scores in the violin plot (Fig. 3c). In stark contrast, GNN-DOVE appears to score all models with very low scores for both the near-native and incorrect classes. Therefore, GDockScore attains state-of-the-art performance and outperforms DeepRank and GNN-DOVE in the highly challenging CAPRI score set.

**Figure 4.**
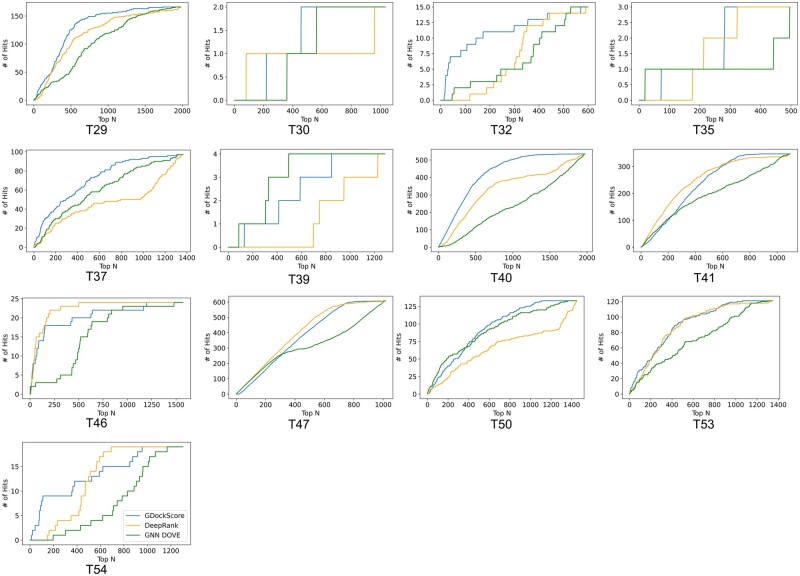
The number of near-native structures (# of Hits) in the top *N* ranked structures for each of the CAPRI score set targets. Here, we display the results of our model in blue, DeepRank in orange and GNN-DOVE in green

Finally, we assessed the model’s performances on each individual CAPRI target by comparing the number of near-native structures in the top *N* ranked structures across a wide range of *N* (Fig. 4). Our model performs comparatively similar to DeepRank and GNN-DOVE across most targets. However, there are many cases where our model appears to outperform DeepRank and GNN-DOVE. Such as examples T29, T32, T37, T40, T53 and T54. This is because our model is putting a larger number of near-native hits in the top *N* decoys at earlier values of *N*. This suggests that our model is, in general, assigning higher ranks to near-native decoys in the CAPRI score set, which is the primary goal of a scoring model for tasks, such as ranking docking decoys. Hit rates (see Section 4) at the top 5 and 10 structures are available in Table 1 to facilitate comparisons to our model. We also compared the number of examples in the CAPRI score set that had a hit in the top 10 ranked decoys for each model. GDockScore had a hit in the top 10 ranked decoys for 6/13 of the unique complexes, GNN-DOVE 7/13, and DeepRank 7/13. For DeepRank, we report 7/13 instead of 6/13 as in Renaud *et al.* (2021) due to a difference in handling tie-breaking when ranking.

**Table 1.** Hit rate % in the top 5 and 10 poses for the CAPRI score set

Target	Top 5 HR %	Top 10 HR %
T29	0.0	0.0
T30	0.0	0.0
T32	0.0	0.0
T35	0.0	0.0
T37	40.0	50.0
T39	0.0	0.0
T40	80.0	80.0
T41	20.0	20.0
T46	40.0	20.0
T47	0.0	0.0
T50	0.0	10.0
T53	40.0	60.0
T54	0.0	0.0

## 3 Discussion

In this work, we present GDockScore, a deep learning-based model that uses protein graph attention to learn a scoring model for protein–protein docking. We generated a new dataset that leverages a substantial portion of the PDB biounits to pre-train GDockScore. We then fine-tuned on a small subset of chains that underwent backbone refinement, with the HADDOCK energy function, or a short molecular dynamics simulation. Our model effectively learns to classify decoys generated by the RosettaDock protocol as acceptable or better according to CAPRI standards, or incorrect. The model also shows promise in the task of distinguishing biological interfaces from crystal interfaces, showing the potential applicability of GDockScore to new protein structure classification tasks.

After this, we sought to challenge our model on the CAPRI score set, a difficult dataset consisting of decoys from many different docking protocols. GDockScore shows better performance compared to other state-of-the-art deep learning models on the CAPRI score set. This includes better AUCROC and AP of the precision–recall curve in comparison to two highly performant models, DeepRank and GNN-DOVE. Upon inspection of the number of hits (near-native decoys) in the top *N* structures, over a complete range of *N*, GDockScore out performed DeepRank and GNN-DOVE in several instances as indicated by having more hits in smaller values of *N*. This is crucial as these scoring models are used to rank decoys and it is optimal to maximize the number of hits in the *N* selected best scoring structures. Our improved model performance on this challenging set can likely be attributed to our pre-training methodology, which utilizes more diverse structures and interfaces, our much finer-grained model features, including information about the positions of all heavy side-chain atoms, and the attention-based model architecture.

There are a variety of ways in which this work can be extended. It is clear that all recent models find the CAPRI score set to be particularly challenging. Similarly to our model having a performance drop from Rosetta decoys to the CAPRI score set when only RosettaDock decoys were used, DeepRank performs much better on classifying the HADDOCK decoys. This drop in performance of models on the CAPRI score set when only one docking protocol is used to generate input data is likely due to the challenging nature of the chain pairs in the set, as well as the variety of algorithms used to generate the decoys. It may be possible to improve the current deep learning models by training on a dataset generated with many different docking algorithms to reduce the likelihood that a model trained on decoys generated by a single docking algorithm is learning the individual idiosyncrasies of the selected algorithm. Another limitation of our model is that our pre-training dataset only includes dimeric complexes. Therefore, the context of the chains interacting in a complex of greater than two chain pairs is lost. Model performance may be improved by training the model to embed complexes of more than two chains at the same time.

In summary, we have developed a new deep learning scoring function for classifying protein–protein docking decoys that performs strongly on decoys generated using the RosettaDock protocol, and the CAPRI score set, a challenging test set in protein–protein docking scoring.

## 4 Methods

### 4.1 Details of the input representation

The input features of the model graphs are based on work developed by Ingraham *et al.* (2019) and Abdin *et al.* (2022). To generate rotationally, and translationally invariant structural features, local coordinate systems are established at each *α*-carbon along the protein backbone allowing for vectors in the global reference frame to be transformed to each protein residue’s local coordinate system, providing rotational and translational invariance.

The node features of the graph include the one-hot-encoded amino acid identity, distances from the *α*-carbon to each heavy side-chain atom raised to a radial basis, directional vectors from each *α*-carbon to each heavy side-chain atom transformed to the local coordinate system, as well as backbone torsional angles embedded in the 3-torus. Our work builds upon Abdin *et al.* by extending the side-chain representation information from a coarse centroid representation to a much more granular all heavy atom representation. Edge features are represented via *α*-carbon to *α*-carbon distances raised to a radial basis, directional vectors transformed to the local coordinate system, as well as quaternion representation of the dot product of the two coordinate systems at each *α*-carbon, which captures information about relative coordinate system orientations. Information about relative distance in sequence was encoded in the way described in the original implementation (Ingraham *et al.*, 2019). Inter-chain edges contain the same information as edges within a protein graph excluding the encoding of relative distance in protein sequence.

### 4.2 Details of the bi-directional attention block

In the bi-directional attention block there are two directions for the update. Suppose there are two proteins, which will be referred to as A and B. The interface residue update for a single interface residue *i* in protein A (*A_i_*) using corresponding neighbour residues in B is as follows: where the query vector $Q_{A_{i}}$ is $R^{1\times d_{k}}$ and generated using a linear layer. The key matrix $K_{A_{i}\to B}$ is $R^{m\times d_{k}}$ and the value matrix $V_{A_{i}\to B}$ is $R^{m\times d_{v}}$, with both being generated using the edges from *A_i_* to the *m* nearest neighbours on protein B. In our model, we have set *m *=* *10. Here, the symbol → indicates that we are updating the protein on the left of the arrow with information from the protein on the right of the arrow. The updates for interface residues in B are generated in a similar manner but using edges from interface residue *B_j_* to the 10 nearest neighbours on protein A. It is also crucial to note that the corresponding node features are concatenated onto the edge features allowing direct exchange of node information from one protein to the other. Thus, if there is an edge from interface node *A_i_* to node *B_j_*, where *i* and *j* are residue numbers in each protein, then the edge *e_ij_* will be concatenated to node embedding *B_j_* before the key value pair generation. Furthermore, in the model implementation all residue updates can be carried out at once by utilizing tensor broadcasting in PyTorch (Paszke *et al.*, 2019). For more details of the generation of the query, key and value matrices please consult Ingraham *et al.* (2019).


(1) $${\text{Attention}}_{A_{i}\to B}(Q_{A_{i}},K_{A_{i}\to B},V_{A_{i}\to B})=\text{softmax}\left(\frac{Q_{A_{i}}K_{A_{i}\to B}^{T}}{{\sqrt{d}}_{k}}\right)V_{A_{i}\to B},$$

### 4.3 Dataset redundancy analysis

The MMseqs2 (Steinegger and Söding, 2017) tool was used to cluster all unique protein sequences in the pre-training set based on similarity with a 30% cut-off point. Furthermore, to ensure that no remaining examples in the training, validation and test sets had any similarity to our intended external test set (Lensink and Wodak, 2014), any structure containing a chain clustered with the CAPRI score set members was removed. As an additional check, the US-align (Zhang *et al.*, 2022) tool was used to calculate TM-scores (Zhang and Skolnick, 2007) to find the structural similarities between the elements of the training, validation and test sets and the targets of the CAPRI score set, with any example with a score ≥0.4 being removed as well, which should eliminate any remotely similar structures (Xu and Zhang, 2010).

### 4.4 Model hyperparameter tuning and training details

Model hyperparameter tuning was performed by varying eight hyperparameters including *d*_model_ (the model embedding dimension), *d*_inner_ (the dimension of the hidden layer in the feed forward layers), *d_k_*, *d_v_*, the dropout percentage, the number of repetitions of the exchange layer, the number of heads in each attention layer and the learning rate. The final tuned parameters were *d*_model_ = 16, number of heads = 3, number of layers = 3, *d_k_* = 16, *d_v_* = 32 and *d*_inner_ = 16.

Training was performed using the Adam optimizer (Kingma and Ba, 2014) with a learning rate of $1\times e^{-4}$, which was determined to be optimal via tuning. The loss function was binary cross-entropy as implemented by PyTorch (Paszke *et al.*, 2019). Early stopping was implemented by monitoring the validation loss during training. The model typically converged at ∼1 epoch of training. Fine-tuning was performed with a learning rate of $1\times e^{-5}$ and the model converged after a little over an epoch of training.

### 4.5 Calculating hit rates

The hit rate is defined to be the total number of cases in which a near-native complex appears in the top *N* decoys. Mathematically this is represented as where $n_{\text{near}\;\text{native}}$ is the number of near-native cases in the current top *N* scorers. Therefore, the hit rate in Figure 4 is simply the hit rate % divided by 100.


(2) $$\text{Hit}\;\text{Rate}\;\%=100\times \frac{n_{\text{near}\;\text{native}}}{N},$$

### 4.6 Definition of AP

The AP reported is from the implementation provided by the scikit-learn machine-learning package (Pedregosa *et al.*, 2011). The mathematical definition is


(3) $$\text{AP}=\sum_{n}\left(R_{n}-R_{n-1}\right)P_{n}.$$

Here, AP represents average precision, the summation is over the *n* thresholds, *R_n_* is the recall at the given threshold, $R_{n-1}$ at the previous threshold and the weighting term *P_n_* is the precision at the current threshold.

### 4.7 Statistical testing

The AUC of the ROC curves was compared using the DeLong test (DeLong *et al.*, 1988) provided by the pROC R package (Robin *et al.*, 2011). The APs obtained from the PR curves were statistically compared using sub-sampling and Welch’s *t*-test. For 1000 sub-samplings, 10% of the data were sampled and used to compute the PR curve and associated AP with scikit-learn (Pedregosa *et al.*, 2011). The difference between the AP for each model was recorded for each sub-sampling. The mean difference in AP between each model was then compared with the Welch’s *t*-test implementation provided by SciPy (Virtanen *et al.*, 2020). A significance value of α=0.05 was used. The Bonferonni correction was used when multiple comparisons were performed.

## Supplementary Material

vbad072_Supplementary_Data

## Data Availability

The dataset introduced in this article is available at http://gdockscore.ccbr.proteinsolver.org. The CAPRI score set files used in this article are available at https://data.sbgrid.org/dataset/684/. The DeepRank dataset is available at https://data.sbgrid.org/dataset/843/. The DC and MANY datasets are available at https://www.eppic-web.org/ewui/#downloads. The code is available at https://gitlab.com/mcfeemat/gdockscore.
