# Supplementary material for: GDockScore: a graph-based protein–protein docking scoring function
Source: Bioinform Adv. 2023 Jun 12;3(1):vbad072. doi: 10.1093/bioadv/vbad072 (PMC10290236; doi:10.1093/bioadv/vbad072)
Supplement: vbad072_Supplementary_Data [file vbad072_supplementary_data.pdf]

A Supplementary Information

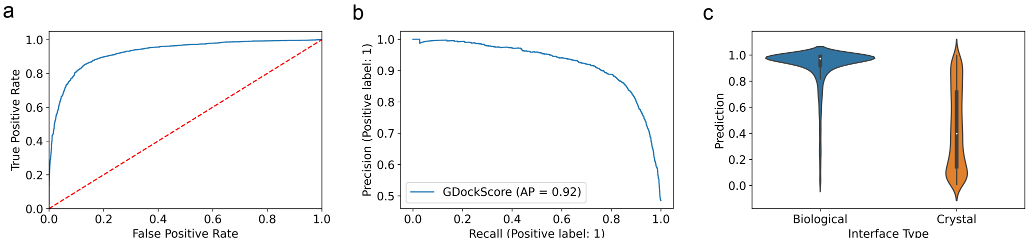

Figure 1: (a) The ROC of the model on the MANY + DC combined data set (AUC = 0.93). The dotted red line indicates pure luck. (b) The precision-recall curve of the model on the MANY + DC combined data set. The inlayed legend contains the AP. (c) Violin plot depicting the distribution of scores for each class of interface (biological vs. crystal).
